# Spatial distribution of prokaryotic communities in hypersaline soils

**DOI:** 10.1038/s41598-018-38339-z

**Published:** 2019-02-11

**Authors:** Blanca Vera-Gargallo, Taniya Roy Chowdhury, Joseph Brown, Sarah J. Fansler, Ana Durán-Viseras, Cristina Sánchez-Porro, Vanessa L. Bailey, Janet K. Jansson, Antonio Ventosa

**Affiliations:** 1 Department of Microbiology and Parasitology, Faculty of Pharmacy, University of Sevilla, Sevilla, 41012 Spain; 2 Biological Sciences Division, Pacific National Northwest Laboratory, Richland, WA 99354 USA

## Abstract

Increasing salinization in wetland systems is a major threat to ecosystem services carried out by microbial communities. Thus, it is paramount to understand how salinity drives both microbial community structures and their diversity. Here we evaluated the structure and diversity of the prokaryotic communities from a range of highly saline soils (EC_1:5_ from 5.96 to 61.02 dS/m) from the Odiel Saltmarshes and determined their association with salinity and other soil physicochemical features by analyzing 16S rRNA gene amplicon data through minimum entropy decomposition (MED). We found that these soils harbored unique communities mainly composed of halophilic and halotolerant taxa from the phyla *Euryarchaeota*, *Proteobacteria*, *Balneolaeota, Bacteroidetes* and *Rhodothermaeota*. In the studied soils, several site-specific properties were correlated with community structure and individual abundances of particular sequence variants. Salinity had a secondary role in shaping prokaryotic communities in these highly saline samples since the dominant organisms residing in them were already well-adapted to a wide range of salinities. We also compared ESV-based results with OTU-clustering derived ones, showing that, in this dataset, no major differences in ecological outcomes were obtained by the employment of one or the other method.

## Introduction

Terrestrial and wetland ecosystems provide critical services of human and ecological importance such as crop production, improvement of water quality through the removal of nitrogen, sequestration of carbon, climate regulation, mitigation of storm surges and support for biodiversity^1–3^. Many of these ecosystem services are carried out by microorganisms. Recent reports of accelerated salinization of coastal wetlands and soils, which poses a significant threat to these ecosystem services, have urged the need to understand the impact of salinity on the structure and diversity of the microbiomes residing in hypersaline terrestrial ecosystems. This knowledge is key for the prediction of ecosystem responses to future environmental changes and for engineering ecological restoration. The few available studies aimed at characterizing the microbial communities in saline soils have mainly focused on either Bacteria^4–7^ or Archaea^8–12^, and less frequently included both domains^13–17^, despite their coordinated contributions to nutrient cycling and soil ecosystem functioning^18^. Therefore, our knowledge of the structure of the microbial communities driving biogeochemical processes in saline soils remains incomplete and fragmented. Moreover, while salinity, along with pH, has been described as one of the most important factors influencing microbial community structure at global and local scales^9,19–22^, analyses carried out in terrestrial hypersaline environments have proposed a secondary role of this factor on microbial assemblages^13,23,24^. Here we aimed to understand the microbiomes of saline soils across different ranges of salinity, to elucidate the main dwellers of these extreme terrestrial habitats and their true relationships with salinity and other soil properties.

We focused on saltmarshes because they represent wetlands at the terrestrial-aquatic interface. Salinity plays a critical role in these environments, affecting ecological functions, including the exchange of material between sediments and seawater and the distribution of plants in the marshes^25,26^. Tidal height and frequency are responsible for the creation of areas with varying salt contents and plant cover, from daily flooded sediments to high marshes that only receive tidal influence once a year^25^. There, interspersed with vegetation, bare soil patches are frequently observed where salinity prevents plant growth. Thus, saltmarshes constitute a great resource for the investigation of salinity influence on soil microorganisms and their distribution in areas with different salinity.

In this study, we selected four high marsh soil locations with extremely high salt concentrations (EC_1:5_ ranging from 5.96 to 61.02 dS/m) within the Odiel Saltmarshes natural area of the southwest coast of Spain, in order to (i) evaluate the diversity, structure and spatial variation of the prokaryotic community and (ii) determine how salinity and other physicochemical characteristics of the soil samples influence the community structure.

## Results

### Soil physicochemical properties

A total of 48 samples were collected and analyzed from 4 sites representing a range of salinities, at two depths (0–1 cm, 2–4 cm). Several characteristics were shared by all samples: they were highly saline, with EC_1:5_ values ranging from 5.96 to 61.02 dS/m, Na and Cl were the main ions, had a low water content (0.08 to 0.32), and showed low elemental concentrations of carbon (5 to 31.9 g/kg), nitrogen (0.12 to 0.87 g/kg), sulfur (0.29 to 14.16 g/kg) and phosphorous (<0.5 to 2.5 g/kg). They also had similar pH values of 7.0 ± 1.3. The contents of Co, Ni, Cr, Cd and Pb were below the detection limit (0.05 mg/kg) in more than 80% of the samples, and very low levels in the rest (Table 1, Supplementary Data S1).

**Table 1:** Measured physicochemical properties of soil samples summarized by sampling site and depth.

Sampling site	1		2		3		4	
Depth	Subsurface	Surface	Subsurface	Surface	Subsurface	Surface	Subsurface	Surface
Nb. of samples	6	6	6	5	6	6	6	5
Water content	0.120 ± 0.005	0.138 ± 0.010	0.292 ± 0.009	0.271 ± 0.015	0.131 ± 0.017	0.155 ± 0.021	0.122 ± 0.018	0.111 ± 0.004
pH	7.287 ± 0.100	7.113 ± 0.079	8.087 ± 0.098	7.914 ± 0.149	7.392 ± 0.194	6.730 ± 0.105	6.843 ± 0.114	6.822 ± 0.037
Carbon (mg/kg)	15,950.000 ± 3001.749	16883.333 ± 2863.846	21350.000 ± 1238.480	18,900.000 ± 1751.285	9933.333 ± 162.380	17,416.667 ± 4009.856	20,950.000 ± 2730.659	22,640.000 ± 3910.703
C:N	52.565 ± 3.634	55.010 ± 1.731	54.134 ± 1.625	55.528 ± 7.603	57.239 ± 8.047	51.447 ± 3.291	46.879 ± 3.081	49.335 ± 5.362
DOC (mg/kg)	1637.483 ± 419.092	2409.683 ± 356.602	2285.250 ± 515.948	4460.400 ± 1017.914	2703.233 ± 2113.304	2407.117 ± 484.233	680.300 ± 268.044	2279.020 ± 239398
EC (1:5 w/v)	15.928 ± 0.990	28.916 ± 3.256	21.098 ± 2.033	50.107 ± 2.327	8.234 ± 0.785	28.696 ± 2.489	9.725 ± 1.151	28.186 ± 3.938
S (mg/kg)	5058.550 ± 1844.487	4354.883 ± 306.323	3579.833 ± 546.724	5879.620 ± 972.326	533.633 ± 88.639	3041.950 ± 265.453	784.067 ± 138.608	3710.520 ± 495.988
K (mg/kg)	488.583 ± 39.423	528.567 ± 59.575	1040.567 ± 109.772	1262.560 ± 163.750	366.283 ± 34.216	1027.383 ± 112.392	514.900 ± 56.696	923.680 ± 63.906
Ca (mg/kg)	1068.933 ± 296.712	1693.950 ± 156.444	967.167 ± 116.587	1324.040 ± 207.970	109.250 ± 27.438	910.633 ± 118.321	155.817 ± 31.734	1077.480 ± 191.394
Na (mg/kg)	13725.833 ± 972.426	30,127.333 ± 3210.893	23,825.333 ± 4806.842	44,392.200 ± 7877.455	7,543.333 ± 788.170	26,904.500 ± 923.358	9139.167 ± 1131.049	24,479.400 ± 3133.966
Nitrate (mg/kg)	<5	<5	2.783 ± 2.783	<5	<5	<5	<5	<5
Sulfate (mg/kg)	9557.667 ± 1156.705	14,980.167 ± 1378.999	13,636.000 ± 2170.485	20,454.600 ± 3586.151	1522.000 ± 302.828	11,301.333 ± 948.068	2286.667 ± 462.485	12,532.600 ± 1491.000
Cl (mg/kg)	26,419.833 ± 2711.620	61,487.500 ± 9708.894	61,482.667 ± 13 906.730	113,532.600 ± 21 827.890	15 513.167 ± 1823.384	63,854.667 ± 3082.933	17,997.667 ± 2404.669	54,833.600 ± 7701.767
Nitrite (mg/kg)	<0.05	<0.05	<0.05	<0.05	<0.05	<0.05	<0.05	<0.05
P (mg/kg)	0.374 ± 0.171	0.524 ± 0.112	0.316 ± 0.225	1.008 ± 0.196	1.312 ± 0.344	1.562 ± 0.123	<0.5	<0.5
Cu (mg/kg)	0.100 ± 0.000	0.474 ± 0.038	0.616 ± 0.420	2.053 ± 0.800	0.117 ± 0.036	0.574 ± 0.120	0.216 ± 0.065	0.539 ± 0.227
Zn (mg/kg)	0.008 ± 0.008	0.075 ± 0.065	0	0.010 ± 0.010	0.383 ± 0.284	0.092 ± 0.027	0.199 ± 0.120	0.160 ± 0.068
Fe (mg/kg)	0.083 ± 0.036	0.083 ± 0.038	0.117 ± 0.065	0.200 ± 0.042	0.208 ± 0.052	1.322 ± 0.668	2.154 ± 0.432	0.888 ± 0.210
Al (mg/kg)	0.258 ± 0.057	0.200 ± 0.013	0.192 ± 0.061	0.359 ± 0.053	0.340 ± 0.120	0.141 ± 0.027	3.562 ± 0.605	1.946 ± 0.434
Mn (mg/kg)	8.172 ± 0.755	7.618 ± 1.287	15.483 ± 1.992	19.488 ± 4.181	4.197 ± 0.515	12.083 ± 1.428	4.270 ± 0.945	15.052 ± 0.962
As (mg/kg)	0.050 ± 0.000	0.042 ± 0.008	0.017 ± 0.011	0.050 ± 0.000	0.050 ± 0.000	0.191 ± 0.035	<0.05	0.020 ± 0.012
Cd (mg/kg)	<0.05	0.058 ± 0.015	<0.05	0.020 ± 0.012	0.008 ± 0.008	<0.05	<0.05	<0.05
Pb (mg/kg)	<0.05	<0.05	<0.05	<0.05	<0.05	<0.05	<0.05	<0.05
Co (mg/kg)	<0.05	<0.05	<0.05	<0.05	<0.05	<0.05	<0.05	<0.05
Ni (mg/kg)	0.008 ± 0.008	<0.05	<0.05	0.010 ± 0.010	<0.05	<0.05	<0.05	<0.05
Cr (mg/kg)	<0.05	<0.05	<0.05	0.010 ± 0.010	<0.05	<0.05	<0.05	<0.05
Mg (mg/kg)	1944.000 ± 156.217	1843.533 ± 247.922	4113.067 ± 627.741	5910.340 ± 1.091.091	863.267 ± 169.525	3346.500 ± 435.200	809.983 ± 241.942	3278.800 ± 446.723

The mean value and standard error of the mean are shown.

They were very diverse in terms of the other measured physicochemical parameters, especially the content in Cu, Zn, Fe and Al, which showed variations of more than 100% across all samples in the dataset, as well as in dissolved organic carbon, DOC (coefficient of variation, CV = 0.95). Specific parameters that significantly differed between the sites were as follows: site 2 had a higher content of water, pH and manganese, and site 4 of iron and aluminium. Concentrations of sulfur, phosphorous and some salinity-related ions (potassium, magnesium, chloride and sulfate) also significantly differed between sites. No measured soil variable was correlated with a particular depth, although there were some differences in conductivity and the content of Na, Ca, K, Mg, Cl, sulphate, sulfur, copper and manganese between surface and subsurface samples within particular sites.

Importantly, some parameters were strongly correlated (r > 0.7, P < 0.05) to each other, which confounded our ability to discern which soil geochemical features were the main drivers of the microbial community structure. Notably, pH and water content measurements were correlated in our dataset (r = 0.7). Conductivity was correlated to K (r = 0.715), Na (r = 0.848), sulfate (r = 0.789), chloride (r = 0.828), Mg (r = 0.722) and Mn (r = 0.715). Calcium was positively correlated to sulfate (r = 0.769) and iron content to aluminium (r = 0.699).

### Microbial community composition

**Figure 1:**
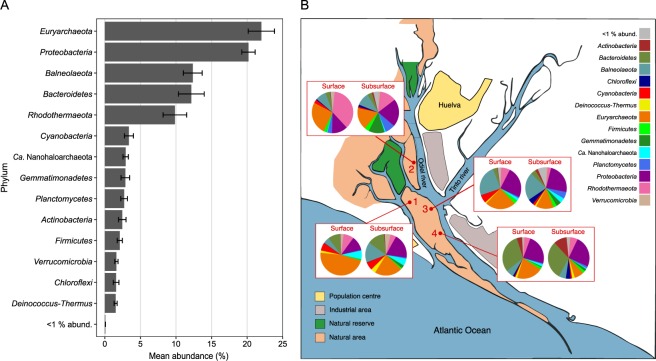
Relative abundances of major phyla detected in the saline soil samples studied. (**A**) Mean abundance of major phyla in the complete dataset. Error bars indicate standard error of the mean. (**B**) Mean abundance values for each site and depth shown over a map of the sampling locations at the Odiel Saltmarshes (Huelva, SW Spain). Abundances are the mean of 6 replicates, except for the surface samples for sites 2 and 4, of which there are 5 replicates.

A total of 429,874 16S rRNA gene sequences grouped into 2809 ESVs (Exact Sequence Variants) and 46 samples passed the quality filtering steps explained in the Methods section. 14 phyla were represented by more than 1% of the total reads in this study. They were: *Euryarchaeota* (3.4–51.5%, with a mean of 22.0 ± 1.9%), *Proteobacteria* (8.8–32.8%, 20.2 ± 0.9%), *Bacteroidetes* (1.2–45.3%, 12.2 ± 1.8%), *Balneolaeota* (1.2–35.2%, 12.4 ± 1.3%), *Rhodothermaeota* (0.8–51.4%, 9.8 ± 1.7%), *Cyanobacteria* (0.3–26.5%, 3.4 ± 0.6%), *Ca*. Nanohaloarchaeota (0.1–8.8%, 2.9 ± 0.4%), *Gemmatimonadetes* (<0.1–16%, 2.9 ± 0.6%), *Planctomycetes* (0.3–12.9%, 2.7 ± 0.5%), *Actinobacteria* (<0.1–15.4%, 2.5 ± 0.5%), *Firmicutes* (<0.1–13.7%, 2.1 ± 0.4%), *Verrucomicrobia* (0.1–7.4%,1.6 ± 0.2%), *Chloroflexi* (0–13.6%, 1.6 ± 0.4%) and *Deinococcus-Thermus* (<0.1–6.3%,1.5 ± 0.2%) (Fig. 1). The 5 most abundant described genera overall, as evaluated by mean abundance, were *Salinibacter* (*Rhodothermaeota*, with a mean abundance of 7.6 ± 1.7%), *Natronomonas* (*Euryarchaeota*; 5.2 ± 0.4%), *Salinimicrobium* (*Bacteroidetes*; 3.9 ± 1.1), *Marinobacter* (*Proteobacteria*; 2.4 ± 0.4%) and *Salinigranum* (*Bacteroidetes*; 2.2 ± 0.3%). No sequences related to the genus *Haloquadratum* were identified. Two uncultured *Balneolaeota* were also among the most represented taxa in this dataset (7.0 ± 0.5% and 3.9 ± 1%).

At the genus level, a total of 170,255 sequences (39.6%) were assigned to uncultured organisms. These sequences were distributed differently among domains: while 92.9% of them were assigned to *Bacteria*, only 7.1% corresponded to archaeal reads.

**Figure 2:**
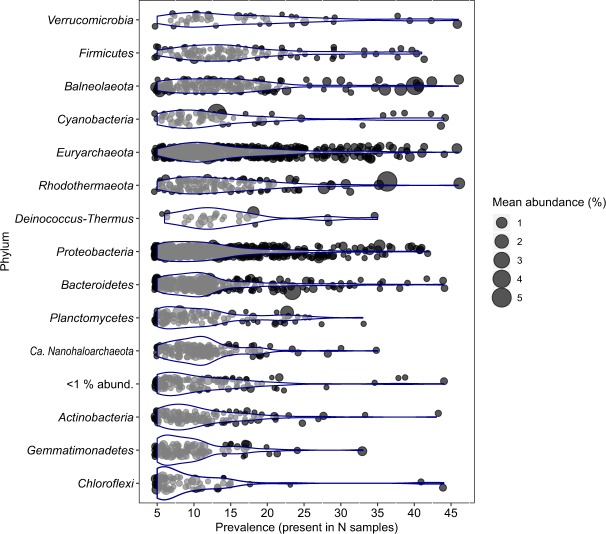
Distribution of ESVs across all samples, grouped by phylum. Points represent individual ESVs and their size indicate their mean abundance in the complete dataset. Phyla are sorted by median prevalence of their ESVs. The distribution of the data and its probability density are shown by violin plots.

Except for *Chloroflexi*, all major phyla represented were detected in all samples. At the ESV-level, only 4 MED nodes, related to uncultured representatives of the phyla *Rhodothermaeota*, *Euryarchaeota*, *Balneolaeota* and *Verrucomicrobia*, appeared to be cosmopolitan (Fig. 2). They had mean relative abundances of 1.0%, 0.7%, 0.6% and 0.4%, respectively. A percentage of 52.6% of ESVs were present in less than 12 samples (maximum number of samples by site considered in this study), and just 61 ESVs affiliated to *Euryarchaeota* (13), *Proteobacteria* (11), *Balneolaeota* (7), *Bacteroidetes* (6), *Cyanobacteria* (6), *Firmicutes* (6), *Verrucomicrobia* (4), *Chloroflexi* (2), *Rhodothermaeota* (2), *Spirochaetes* (2) as well as *Actinobacteria* (1) and *Kiritimatiellaeota* (1), were found in more than 36 samples (number corresponding to 3 sites). Three sequence variants were especially abundant, comprising more than 4% of the reads by mean. They were affiliated to uncultured representatives of *Balneolaeota* (with a prevalence of 40 and mean abundance of 4.1%), *Rhodothermaeota* (with a mean abundance of 5.3% and a prevalence of 36), as well as to the cyanobacteria *Phormidium* (mean abundance of 4.4% and identified in 13 samples).

Indicator species analysis calculated according to Dufrêne-Legendre method extended by De Cáceres, which considers fidelity and exclusivity, revealed that 62,7% of ESVs were indicative of a specific sampling location. Sampling site 4 harbored the higher number of indicator variants (635, 22.6% of the total), followed by site 1 (523, 18.6%), 3 (348, 22.6%) and 2 (255, 12.4%). 17.7% and 9.1% of the total number of ESVs were indicative of 2 and 3 locations, respectively. These results were significant at α = 0.05.

### Alpha diversity metrics and correlation with soil properties

Alpha diversity analysis showed that, while subsurface samples (2–4 cm) were not different among sites in terms of Shannon and Simpson indices, the diversity of surface samples (0–1 cm) varied with sampling location (Supplementary Figure 1). Alpha diversity was different in surface and subsurface samples from site 1 (according to Simpson index) and site 2 (according to both Shannon and Simpson indices) (P < 0.05). The calculated indices were significantly correlated to calcium content (r = −0.512 for Shannon and r = −0.501 for Simpson index, at α = 0.05).

### Relationship between soil properties and microbial community structure

Ordination analysis based on the Bray-Curtis dissimilarity index was performed in order to assess the relationship between samples’ prokaryotic community structure. It showed that samples clustered by sampling site. A secondary cluster could be observed for samples from the same depth within a site. The environmental variables fitted onto the ordination analysis indicated that soil properties such as sand proportion, water content, pH, total sulphur and phosphorus, sulphate, calcium, magnesium, conductivity, aluminium and iron were significantly correlated to samples separation. PERMANOVA analysis confirmed that the correlation of these parameters with community structure variation was statistically significant (P < 0.05), and that the factors “site of sampling” and “depth” explained 24.4% and 4.6% of the variance in community composition, respectively.

**Figure 3:**
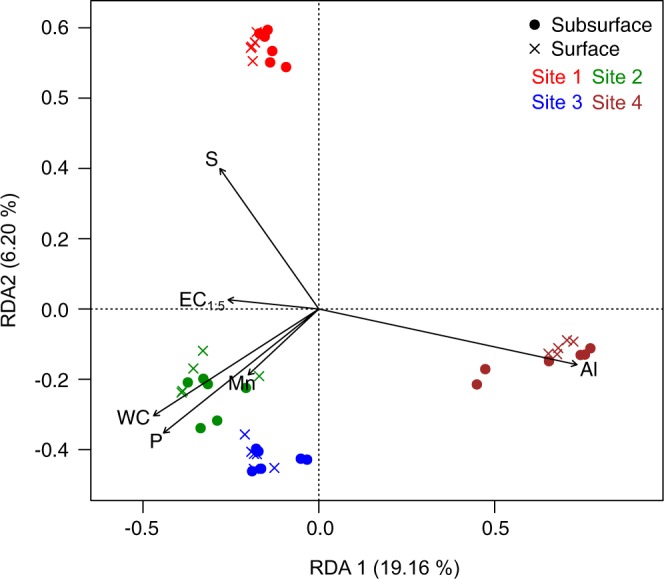
RDA ordination plot including the soil variables that best explain community data, as determined by forward selection. It is based on Hellinger-transformed abundance data. It was significant at α = 0.05 according to Monte Carlo permutation tests. S: total sulfur, EC_1:5_: electrical conductivity measure in an 1:5 w/v soil to water extract, WC: water content, P: phosphate, Mn: manganese and Al: aluminium.

In order to evaluate the continuous soil variables that best explained community structure, we carried out RDA analysis with forward selection model on the data after selecting non-correlated soil parameters (that is, after removing iron, salinity-related ions and texture associated parameters). The best explanatory model included the variables aluminium, water content, phosphorous, sulfur, conductivity and manganese (Fig. 3), had a variance inflation factor (VIF) below 3 for all considered parameters, and was statistically significant at α = 0.05. This set of environmental variables explained 39.7% of the variance in community composition. Evaluation of partial effects of predictors revealed that aluminium alone was responsible for a 9.5% of the variation in community composition, while phosphorous, water content, sulfur, conductivity and manganese explained 7.5, 7.3, 5.2, 3.0 and 2.8% of the differences in community structure among samples, respectively. “bioenv” test (*vegan*) showed that, among all considered physicochemical variables, it was aluminium content that best correlated to shifts in community composition (r = 0.520).

**Figure 4:**
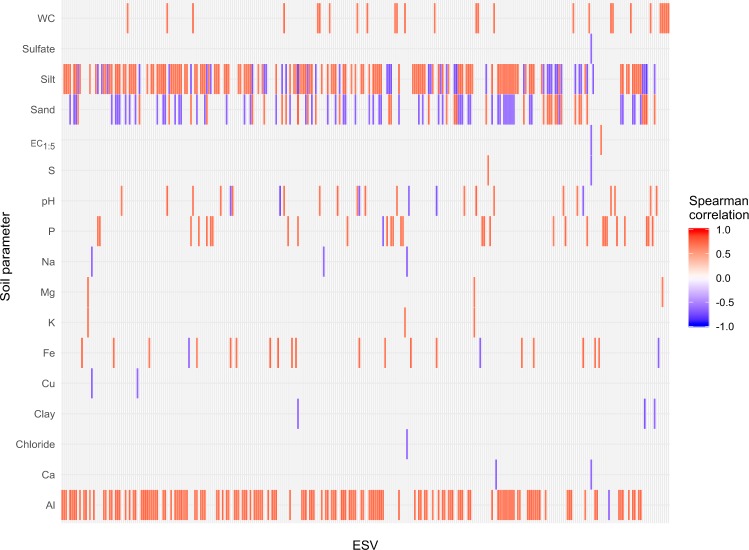
Correlation of soil parameters to ESVs relative abundance. All shown correlations are significant at α = 0.05. The complete list of correlation coefficients and ESVs identity and taxonomy from this plot can be found in Supplementary Data S2.

The correlation of the relative abundance of ESVs with measured physicochemical parameters showed that 19.7% of the total number of ESVs were associated to at least one of the measured soil properties. A total of 151 ESVs were correlated with aluminium concentration, 177 with silt and 97 with sand content. Other soil parameters associated with particular ESVs abundances were water content, sulfate, conductivity, sulfur, pH, phosphorous, sodium, magnesium, calcium, iron, copper, clay, chloride and calcium (Fig. 4). Taxonomy assignation of these ESVs and their Spearman correlation value with soil properties can be found in Supplementary Data S2. Cosmopolitan ESVs detailed above did not correlate to any measured physicochemical soil feature.

### Comparison of ESV and OTU-clustering approaches

ESV-based binning approaches enable finer taxonomic resolution and may be able to discern between strains with different environmental preferences that OTU-based approaches would not be able to detect^27,28^. However, it has been shown that this method does not always provide different ecological outcomes from traditionally used OTU clustering at 97% sequence similarity^29^. Aiming at assessing the performance of each of these widely used binning methods in revealing ecological insights in this dataset, we also analyzed the data at the OTU level and compared the results with ESV-based ones. A total of 507,568 reads passed the quality filtering and grouped in 2,771 OTUs. Evaluation of prevalence in the studied soils revealed that 15 OTUs were detected in all samples. Five of them were related to the phylum *Balneolaeota*, while the rest were affiliated to *Proteobacteria* (4), *Euryarchaeota* (3), *Rhodothermaeota* (1), *Cyanobacteria* (1) and *Verrucomicrobia* (1). Except for two *Proteobacteria* OTUs, which were assigned to different species of the genus *Marinobacter*, the rest of them were related to uncultured representatives from these taxa.

At this level, 41.6% OTUs were indicative of one sampling location according to IndVal index. 477 OTUs were identified as indicators in site 4, 282 in site 1, 253 in site 3 and 141 in site 1.

With Shannon index ranging from 3.22 to 5.95, and Simpson’s from 0.82 to 0.99, trends observed for alpha diversity metrics between sites and depths were the same as those indicated above for ESV-based analysis. In this case, Shannon index was negatively correlated with content in Cu (r = −0.58), Na (r = −0.57), Cl (r = −0.57), K (r = −0.53), DOC (r = −0.52), conductivity (r = −0.51) and Mg (r = −0.47). Simpson index values decreased with content in K (r = −0.53).

Procrustes analysis shows that OTU-based clustering of samples in NMDS (Non-Metric Multidimensional Scaling) ordination is similar to that calculated at the ESV level (Supplementary Fig. S2). Site, water content, sand, silt, conductivity, sulfur, Ca, Na, sulfate, Cl, aluminium and Mg were correlated to community composition according to “envfit” calculation on NMDS ordination. Forward selection of predictors based on redundancy analysis (RDA) revealed that Al, water content, phosphorous, conductivity, sulfur and manganese were the measured variables that best explained community structure variation. Together, they were responsible for 40.3% of the differences in community structure among samples. Aluminium content was identified as the variable best correlated to community data according to “bioenv” function in *vegan*.

Only 12.1% of the total number of OTUs were correlated with environmental variables: 3.4% of OTUs were associated with silt content, 3.4% to aluminium concentration and 2% to clay content. Less than 1% of OTUs were significantly correlated to Ca, clay, Cu, Fe, K, Mg, Na, P, pH, S, conductivity, sulfate and water content.

## Discussion

Some of the dominating phyla in these soils, such as *Euryarchaeota*, *Proteobacteria, Bacteroidetes* and *Rhodothermaeota* (Fig. 1A), have already been shown to be among the main taxa found in other saline soils^4,6,7,10,13,14^ and in a previous study of saline soils from this same location consisting on a metagenomic snapshot of the microbiome from two time points^17^. These are also among the most abundant groups present in widely studied aquatic hypersaline environments^7,20,30–37^. However, the phylum *Balneolaeota*, which was especially represented in site 3 (Fig. 1B), has only been reported as abundant in saline soils from the Odiel Saltmarshes^17^. The most abundant genera within these phyla consisted of known halophiles. *Natronomonas* was among the most abundant euryarchaeal genera represented in these soils, in agreement with previous reports of the archaeal communities from other studied saline soils^10,14,15^. We noted the absence of reads affiliated to the genus *Haloquadratum*, which constitutes more than 75% of the community in the highest salinity saltern ponds from Bras del Port saltern (Santa Pola, Alicante, Spain), one of the best studied hypersaline ecosystems^30^. However, this genus also seems to be missing from the microbiota of other saline soils^9,10,12^, and was not detected in previous metagenomic studies from saline soils from this same area^17^. Thus far, only two studies have retrieved sequences related to this genus, albeit in low abundance, in saline soil samples from the desert Rann Kutch^14,15^ (Gujarat, India). On the other hand, the genus *Salinibacter*, which is commonly found in hypersaline waters^38^, shares habitat with *Haloquadratum* in crystallizer ponds^39^, and has also been reported in saline soils before^14,15^, was the most abundant bacterial genus overall, followed by *Salinimicrobium*. One environmental genome related to the latter genus was extracted from metagenomes of samples collected in the same area^17^, which suggests that organisms affiliated to *Salinimicrobium* may be relatively abundant in these soils over time. Other bacterial genera detected in high abundance included *Salinigranum* and the nutritionally versatile *Marinobacter*.

We also detected sequences affiliated to *Ca*. Nanohaloarchaeota, an archaeal candidate taxon which was first found in intermediate salinity aquatic habitats^30,40^ and that has since been detected in salt-saturated habitats^41^ and halite endoliths^42^. To our knowledge, the only record of its presence in saline soils to date is the previous study performed on the Odiel Saltmarshes soils^17^. Among the other phyla shared by all samples, *Cyanobacteria*, *Actinobacteria*, *Firmicutes* and *Verrucomicrobia* are known to contain true halophilic or halotolerant species, while *Gemmatimonadetes*, *Planctomycetes*, *Chloroflexi* and *Deinococcus-Thermus* have been detected in saline soils before, yet none of their members has been described to require more than 1% of NaCl to grow^43,44^. The latter is also the situation for the rest of the groups detected in these soils in low abundance^44,45^. Although the presence of taxa without known halophilic representatives in soils with very high salt contents may be surprising, several situations may explain this result. The heterogeneity of the soil matrix and the low water content of these unsaturated soils may allow for niches with variable abiotic parameters (such as salinity) to co-exist and be poorly connected^46–49^, hence providing shelter and/or suitable growth conditions for non-halophilic microorganisms^10^. Furthermore, we cannot reject the possibility that the untapped diversity from these groups could harbor true halotolerant or halophilic microorganisms awaiting to be described.

Since almost all major phyla were cosmopolitan (albeit differentially represented as can be observed in Fig. 1B), we evaluated the possibility that phylotypes from these groups may also be detected in all samples. Fig. 2 shows the number of samples in which each ESV is present, as well as their mean abundances in the dataset. At this finer level of resolution, most sequence variants (52.6%) were present in less than 12 samples (number of samples by site of sampling), 2,2% of the total number of ESVs were detected in more than 36 samples (~3 sites), and only 4 were cosmopolitan. They were related to uncultured representatives of the phyla *Rhodothermaeota*, *Euryarchaeota*, *Balneolaeota* and *Verrucomicrobia* and their mean abundances ranged from 0.47 to 1.01%. While not detected in all samples, 3 sequence variants groups comprised more than 4% of the reads by mean in the dataset. Taxonomic annotation of these variants revealed that the ones related to *Balneolaeota* and *Rhodothermaeota* were not closely related to cultivated strains from these phyla. The third ESV with a very high mean relative abundance was related to *Phormidium* (*Cyanobacteria*), was present in 13 samples and had a mean relative abundance of 4.4%.

It can be assumed that ESVs identified in all samples with high mean abundances are prone to be successful in most of the samples and thus, may be versatile enough to cope with the ranges in nutrients and environmental conditions covered in this study. Therefore, our data highlight that some of the most abundant, successful and cosmopolitan organisms in the studied soils remain uncharacterized. The high number of sequences affiliated to uncultivated microorganisms at the genus level, as well as the high abundance and prevalence of some of them, emphasizes the effort still needed in order to comprehensively characterize the microbiota from this type of hypersaline habitats, and especially their bacterial representatives.

While some prokaryotes of these soils may be ubiquitous and successful in the range of physicochemical conditions that the studied samples encompass, the prevalence results as well as indicator species analysis showed that 62.7% of the sequence variants could be identified as indicative of a particular sampling site, which suggest that specific properties of the soil samples included in this study may be restricting factors for most dwellers and probably have a role in determining the microbial community across the dataset. The NMDS ordination analysis based on Bray-Curtis dissimilarity matrix and complementary statistical tests showed a main clustering of samples by site of sampling. This result is consistent with PERMANOVA tests, which showed that “site of sampling” explained 24.4% of the variance in community composition, and “depth” was associated with a 4.6% of the shifts in community structure. Thus, although the high salt content in all samples determined the halophilic nature of the major groups of inhabitants, site-specific soil characteristics defined community structure in each sample. We further investigated the physicochemical parameters susceptible of affecting the microbial community composition in the ranges considered in this study. Vector fitting analysis on NMDS ordination indicated that sand proportion, water content, pH, total sulphur and phosphorus, sulphate, calcium, magnesium, conductivity, aluminium and iron were correlated with prokaryotic community composition, and aluminium content was identified by “bioenv” function (*vegan*) as the best factor correlating to community structure. Analysis based on RDA showed that the best predictors of the community composition (from the subset of non-correlated soil variables) were aluminium, water, phosphorous, sulfur and manganese content, as well as conductivity. Together they were responsible for approximately 40% of the variance in community structure among samples. These parameters were significantly different among sites, but not all properties significantly different among sites were identified as contributors to the observed shifts in the microbial community. The association of water content, phosphorous and sulfur with prokaryotic community structure in saline soils have been reported before^6,13,50,51^. Metal concentrations, which we measured because of their high load in sediments from the Odiel Saltmarshes in previous studies^52–55^, did not exhibit elevated values in the measured soils and did not surpass the legal limits for considering them as contaminated. Except for aluminium and iron, for which the higher contents in site 4 were partly responsible for its separation from the rest of sites in the NMDS plot, no other metals were shown to have an impact on the structure of the microbial community in this dataset; despite salinity having previously been shown to contribute to metal mobilization and thus, to the modulation of their bioavailability and toxicity^56,57^. Evaluation of the correlation of soil physicochemical features with ESVs abundances showed that aluminium, together with texture-related variables, was among the parameters most associated with changes in variants proportions in each sample (Fig. 4).

All analysis carried out point towards aluminium content being an important contributor to the variance in community composition in the studied dataset. To our knowledge, the impact of aluminium on microbial communities from habitats with near-neutral pH such as the ones considered in this study has not been previously described. Aluminium, a widely abundant element on Earth’s crust that is not known to participate in biological processes^58,59^, has a complex chemistry. Its speciation is governed mainly by pH, such that its bioavailability increases with decreasing pH, but other factors like ionic strength or concentration of inorganic compounds and chemical complexing agents also influence this process^58–60^. We hypothesize that the putative effects of aluminium in the microbial community structure from these soils may be driven by the influence of other components on its solubilization. However, it is worth noting that aluminium and iron are correlated in this dataset, which difficults the determination of their individual contributions to microbial community composition variance.

Evaluation of within-sample diversity at the ESV level revealed a rich and evenly distributed prokaryotic community in the studied soils. The values of Shannon and Simpson indices were just weakly correlated to calcium content. These results suggest that other unmeasured soil variables may be influencing the diversity (and possible also structure) of the microbiome of these hypersaline soils.

In this study, salinity (assessed as the electrical conductivity of 1:5 w/v soil water extracts) was found as a predictor of observed changes in the community by forward selection based on RDA ordination, but it was just responsible for a 3% of the variation in community structure. It was not related to richness or evenness of the prokaryotic community. Furthermore, just two ESVs were significantly correlated, positively or negatively, to conductivity in our dataset. These results suggest that the influence of soil salt content on the microbiota of these highly saline soils is limited, probably due to the high grade of specialization to life at elevated and changing external salinity of their main dwellers. Additionally, the threshold of salinity that impacts community structure may be lower than the range of samples we studied. Although salinity is considered as one of the main drivers of microbial community structure^9,19,22,61^, this is not the first report in which the critical influence of this parameter in microbial community is not observed^13,23,24^. Hollister *et al*. proposed that, once a certain salinity threshold is surpassed, salt content would exert a weaker influence since most of the organisms present would already be either halophilic or halotolerant, or would be able to survive in these extreme conditions^13^. Considering that the least saline sample in this study is highly saline (EC_1:5  _=5.96) and that the microbial community was mainly composed of microorganisms adapted to life at high salt concentrations, our results support that hypothesis. Furthermore, halophilic microorganisms retrieved from terrestrial habitats have been shown to be especially flexible in the range of external salinities they can thrive in^11,62–64^, and the influence of salinity on community composition may be weaker in soil habitats than in aquatic ones^7^. The fact that just 2 sequence variants, from a total of 2809, correlated to conductivity (Fig. 3), and the high prevalence of some phylotypes across the samples of this study (Fig. 2), further corroborate the proposed euryhaline character of halophiles thriving in soil. Therefore, we suggest that the level of salinity considered in a particular study, along with the nature of the habitat (soil *vs* water) may be key factors when integrating results of the influence of salinity in microbial community structure from different investigations.

Overall, these soils harbored a unique microbiota, indicating that their further study may provide new insights about the physiology and ecology of taxa without environmental information to date, and foster the isolation of new uncharacterized microorganisms. Our findings support the hypothesis that salinity thresholds influence the composition of soil microbiomes, with halophilic species abundant in all samples. Furthermore, the differences in community composition and structure among our samples is largely attributable to soil-specific properties, such as aluminium, water content, phosphorous, conductivity, sulfur and manganese concentrations. This suggests that even under highly restricting conditions, native soil properties continue to influence the microbiome in tandem with stressors such as salinity.

The discussed results, which derived from the ESV-based analysis, are similar to the ones obtained through OTU-based clustering methods. Although the higher resolution achieved by MED allowed to discern among ecotypes with different environmental and/or nutritional preferences that were lumped at the OTU level (as revealed by the lower number of cosmopolitan groups and higher proportion of phylotypes correlated to soil variable detected in the ESV-based analysis), still abundant and cosmopolitan organisms were identified in both cases and aluminium and texture were the variables with higher number of correlations with particular ESVs/OTUs. Alpha and beta-diversity trends were also equivalent. Although alpha diversity metrics were correlated with different parameters in each approximation, in neither case the main driver of richness or evenness was identified among the measured variables. Therefore, in this dataset, no major differences in ecological outcomes were observed by the employment of one or the other method.

## Methods

### Site description

The Odiel Estuary Saltmarshes are located at the joint estuary of the Odiel and Tinto rivers (Huelva, Southwest Spain). These marshes are protected as a UNESCO Biosphere Reserve and a RAMSAR site. This Atlantic estuary is located in an area with a Mediterranean climate, with a dry season during the summer and frequent rainfall in autumn and winter. Mean annual precipitation is 506 mm. The mean, maximum and minimum temperatures reported are 18.3, 31.6 and 7.7 °C, respectively. The average tidal range is 2.10 m in a semidiurnal regime, high tides reaching almost 4 m during equinox^65,66^. Tidal cycles create distinct habitats within marsh creeks in saturated (low), intermittently flooded (medium), and unsaturated (high) zones. The most common vegetation include halophytes such as *Spartina densiflora, Salicornia ramossisima* and *Sarcocornia*^65^. Draining the Iberian Pyrite Belt, the Odiel and Tinto rivers transport a load of metals (Al, Zn, Fe, Mn, Cu, Co, Cd, Pb, Cr, Ni, As) through the salt marsh area, which is also affected by industrial effluents from the nearby factories^67,68^.

### Soil sample collection

Soil samples were collected in July 2015 from high marsh areas that are entirely rain-fed and experience just occasional high tide events. Four sampling sites were selected where areas of bare soil alternate with *Salicornia*-covered zones (Fig. 1B). Within each site, several sampling points separated by 2.5–6 m, and two depths (surface: the upper centimeter, and subsurface: up to 2–4 cm) were sampled in 4 oz. Whirl-Pak bags. Average soil temperature recorded at depth 1 cm was 43.5 °C. Samples were transported on ice in the dark to the University of Sevilla and stored at −80 °C until shipping on dry ice to the Pacific Northwest National Laboratory (PNNL), Richland, USA. We randomly selected samples comprising 6 samples from each site and depth (treated as biological replicates) for this analysis.

### Physicochemical characterization of soils

Sieved (<2 mm) soil samples were analysed for soil properties. Wet soil subsamples were employed for salinity characterization by measuring electrical conductivity in 1:5 w/v water extracts (EC_1:5_) with conductimeter CRISON 35+ (Hach Lange Spain, S.L.U.), which corrects for temperature automatically. Air-dried soil was used for other determinations. pH measurements were also carried out in 1:5 w/v water extract using pH meter CRISON GLP 21. Water content was determined gravimetrically by drying to constant weight at 100 °C overnight. Innoagral (Grupo Hespérides Biotech S.L., Sevilla, Spain) performed the analysis of texture, total carbon (TC), total nitrogen (N), total sulphur (S), total phosphorus (P), dissolved organic carbon (DOC), total anions (sulphate, chloride, nitrate and nitrite) and the following cations: potassium (K), calcium (Ca), magnesium (Mg), copper (Cu), zinc (Zn), aluminium (Al), arsenic (As), cadmium (Cd), lead (Pb), iron (Fe), cobalt (Co), nickel (Ni), manganese (Mn) and chromium (Cr). Texture was analysed by the Bouyoucos method^69^. Carbon was determined by Walkley & Black^70^ and total nitrogen by Kjeldahl method^71^. Ion Chromatography (850 Professional IC Anion – MCS, Metrohom AG, Switzerland) was used to measure nitrate, nitrite and sulphate^72,73^. Chloride content was measured by volumetry^74^. iCAP^TM^ 6500 Duo ICP-OES (Inductively Coupled Plasma – Optical Emission Spectrometer) analyzer (Thermo Fisher Scientific) was employed for determining all other analytes^75^. Extractions were performed from 10 g of dry soil in the proportion 1:5 w/v. Ions were measured from water extracts and metals were analysed from nitric acid 2% extract^75^. Except for analytes determined by ICP-OES, for which the detection limit was 0.05, the detection limit was set to 0.01.

Data for physicochemical variables that were below or at the detection limit of analysis (such as nitrate, nitrite, arsenic, cadmium, lead, cobalt, nickel and chromium) for ≥80% of the samples were not used in the analysis to avoid spurious correlations. Students t-tests were performed to detect differences in properties between the surface and subsurface samples from each site. Statistically significant differences among sites were determined using ANOVA test followed by Tukey HSD test^76^, after normality of the data had been checked with Shaphiro-Wilk test. Pearson correlations were used to evaluate the relationships between individual physicochemical parameters and between these and alfa diversity metrics.

### DNA extraction and amplicon sequencing

Soil samples were briefly thawed and homogenised by wet sieving through pre-sterilized 2-mm mesh screens. Soil processing was carried out in a cold room (4 °C). Sieved samples (0.25 g) were washed (3 times) using 1X sterile Phosphate Buffered solution in a ratio 1:4 w/v.

DNA was extracted using the PowerSoil DNA Isolation kit according to manufacturer’s instructions (MoBio Laboratories Inc., Carlsbad, CA). DNA integrity was checked on a 3% agarose gel, and its quantity and quality were assessed using Qubit 2.0 fluorometer (Thermo Fisher Scientific), Nanodrop 2000 (Thermo Scientific) and Picogreen assay (Thermo Fisher Scientific).

The V4 region of the 16S rRNA gene was amplified using the PCR protocol developed by the Earth Microbiome Project (http://press.igsb.anl.gov/earthmicrobiome/emp-standard-protocols/16s/)^77^. This protocol was modified such that the 12 base barcode sequence was included in the forward primer. Amplicons were sequenced on an Illumina using the 500 cycle MiSeq Reagent Kit v2 (http://www.illumina.com/) according to manufacturer’s instructions. In the sequencing process, PhiX in a low concentration spike-in was used to monitor sequencing quality control. Controls without template, as well as positive ones, were included to ensure amplicon generation was not contaminated during the protocol.

### Community and diversity analysis

The program sdm (simple demultiplexer)^78^ was used for demultiplexing. VSEARCH^79^ v2.3.0 was then employed for quality filtering and dereplication. Resulting sequences were trimmed to a uniform length of 254 nt prior to the following analysis. Exact sequence variants (ESVs) were obtained by using Minimum Entropy Decomposition (MED) algorithm. Minimum substantive abundance of an oligotype or MED node (-M) was set to 12 as previously recommended^80^. The minimum variation allowed in each node was set to 3 by default. For OTU binning process, *de novo* and reference-based chimera checking and clustering at 97% similarity were performed with VSEARCH. Taxonomic assignment was conducted with assign_taxonomy.py from QIIME v1.9^81^ using SILVA database^82^ v.132 and followed by manual curation in order to update phylum-level annotation of sequences belonging to *Rhodothermia* and *Balneolia* from their older phylum-level assignation (*Bacteroidetes*) to *Rhodothermaeota* and *Balneolaeota* phyla, respectively^83,84^.

Downstream analysis was carried out in R v3.2.0^85^. The package *phyloseq* v.1.14.0^86^ was employed to remove samples with less than 3500 sequences, those not matching the domains *Bacteria* or *Archaea*, as well as OTUs or ESVs present in less than 5 samples (minimum number of replicates available in this study). Mean abundances of phyla and genus were calculated based on relative abundances. Graphical representations were carried out with *ggplot2* v2.2.0^87^. The package *indicspecies*^88^ was used to perform Dufrêne-Legendre indicator species (ESV or OTU) analysis^89^ of different sites. Significance was set at α = 0.05.

To account for different sequencing depths, the ESV/OTU table was normalised to the upper 75^th^ quartile using *DESeq2* v.1.22.1^90^ before evaluation of within- and among groups diversity (α- and β-diversity) at the ESV/OTU level with *vegan* v2.4.1^91^ package. Differences in α-diversity across groups were evaluated using Wilcoxon tests^92^, for comparing the two depths, and Kruskall-Wallis^93^ followed by Wilcoxon tests, for sites. To assess the relationship of the soil properties with α-diversity indices (Shannon and Simpson), Spearman correlations were carried out. Soil variables with not normal distribution were log-transformed. Bonferroni correction was applied to resulting P values and significance level was set at α = 0.05. For comparing the microbial community structure among samples, Bray-Curtis dissimilarity index was calculated for each pair of them, and the resulting matrix subjected to non-metric multidimensional scaling (NMDS) ordination analysis carried out using “metaMDS” function (*vegan*). Vector fitting of environmental parameters to this ordination (with “envfit” function within the *vegan* package) allowed the evaluation of the relationship of each of them with the community composition. To corroborate those relationships and assess their significance, PERMANOVA analysis (“adonis” function in *vegan*) was performed. Comparison between ESV- and OTU-based NMDS ordinations was carried out by Procrustes analysis in *vegan*. Significance was tested by Monte Carlo simulations. RDA ordination with forward selection was carried out in *vegan* with Hellinger-transformed raw abundance data in order to determine the variables that best explained variance in community structure. Significance was tested by permutation tests. Partial effects of predictors were calculated through conditional ordination analysis. “bioenv” function (*vegan*) was used with Bray-Curtis dissimilarity matrix to determine the subset of environmental variables in the analysis that best correlated to community data from the set of measured ones. Spearman correlations were performed to evaluate the relationship between the soil measured physicochemical factors and individual sequence clusters relative abundance.

### Nucleotide sequence accession numbers

Data have been deposited in the SRA under BioProject PRJNA378479, with individual accession numbers listed in Supplementary Data S1.

## Supplementary information

Supplementary Figures

Dataset 2

Dataset 1
