# Supplementary material for: Spatial distribution of prokaryotic communities in hypersaline soils
Source: Sci Rep. 2019 Feb 11;9:1769. doi: 10.1038/s41598-018-38339-z (PMC6370769; doi:10.1038/s41598-018-38339-z)
Supplement: Supplementary file 1 — Supplementary Figures [file 41598_2018_38339_MOESM1_ESM.pdf]

Spatial distribution of prokaryotic communities in hypersaline soils

Blanca Vera-Gargallo[1], Taniya Roy Chowdhury[2], Joseph Brown[2], Sarah J. Fansler[2], Ana Durán-Viseras[1], Cristina Sánchez-Porro[1], Vanessa L. Bailey[2], Janet K. Jansson[2] and Antonio Ventosa[1]*

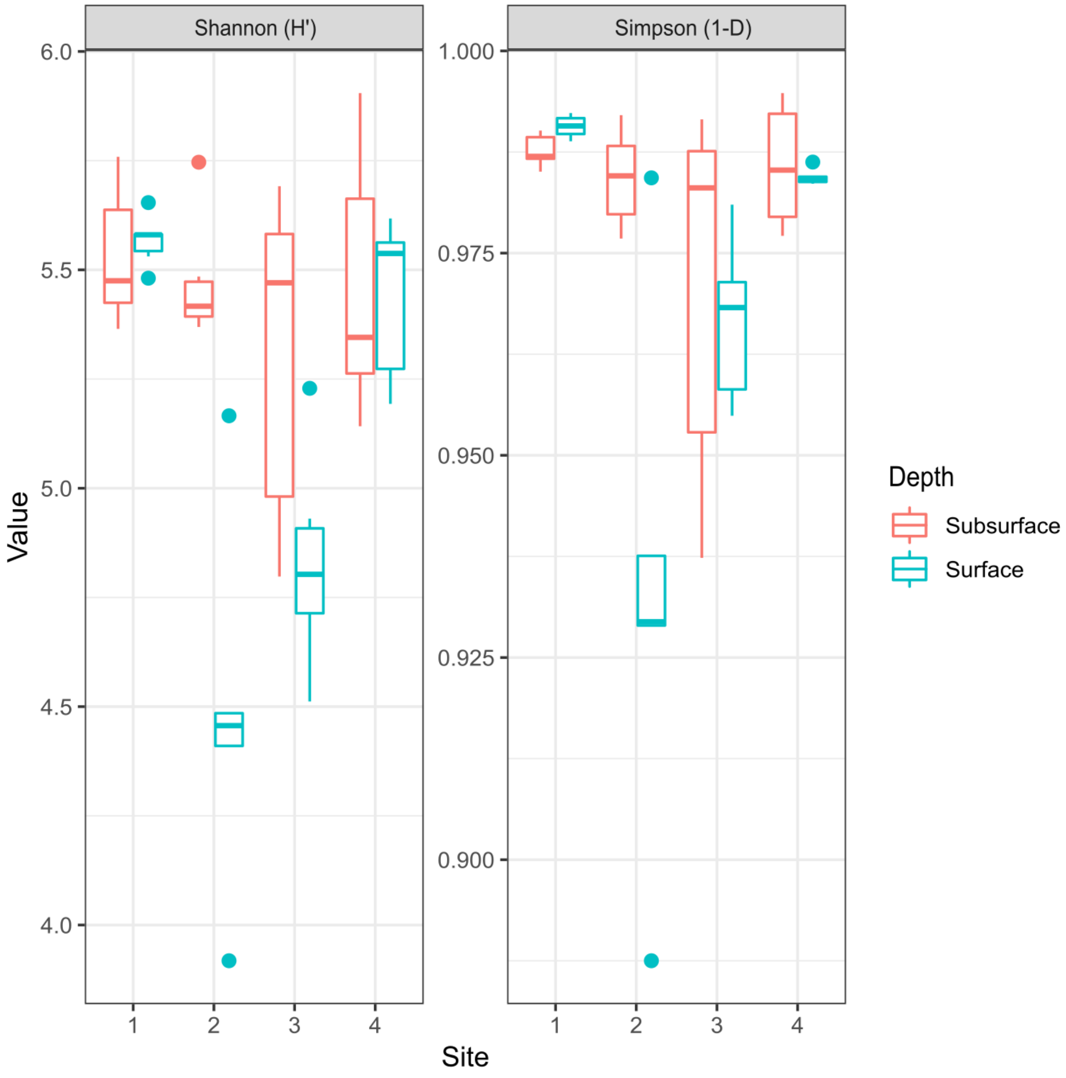

Supplementary Figure S1. Alpha diversity metrics (Shannon and Simpson indices) for each site and depth calculated at the ESV level. The plot displays the median, upper and lower 25% quartiles, minimum, maximum and outliers of alpha diversity values.

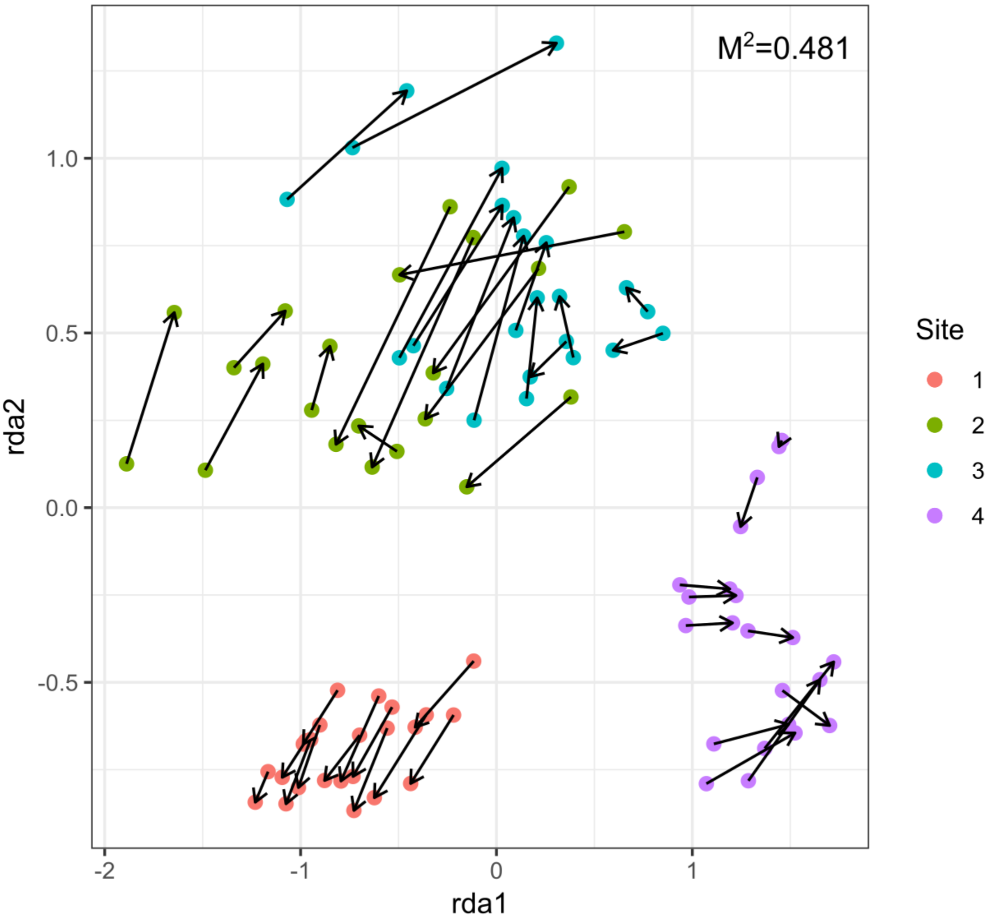

Supplementary Figure S2. Procrustes analysis of NMDS ordination analysis based on ESVs and OTUs. NMDS ordination was performed on Bray-Curtis distances of normalized abundance data from ESV-based and OTU-based analyses. Arrows start on ESV-based NMDS data and end on OTU-derived data. The fit of procrustes transformation is indicated as M^2.
